# Delays and Factors Related to Cessation of Generalized Convulsive Status Epilepticus

**DOI:** 10.1155/2015/591279

**Published:** 2015-08-10

**Authors:** Leena Kämppi, Jaakko Ritvanen, Harri Mustonen, Seppo Soinila

**Affiliations:** ^1^Clinical Neurosciences, Neurology, University of Helsinki and Helsinki University Hospital, 00029 Helsinki, Finland; ^2^Department of Surgery, Helsinki University Central Hospital, 00029 Helsinki, Finland; ^3^Division of Clinical Neurosciences/General Neurology, Department of Neurology, Turku University Hospital, University of Turku, 20521 Turku, Finland

## Abstract

*Introduction*. This study was designed to identify the delays and factors related to and predicting the cessation of generalized convulsive SE (GCSE). *Methods*. This retrospective study includes 70 consecutive patients (>16 years) diagnosed with GCSE and treated in the emergency department of a tertiary hospital over 2 years. We defined cessation of SE stepwise using clinical seizure freedom, achievement of burst-suppression, and return of consciousness as endpoints and calculated delays for these cessation markers. In addition 10 treatment delay parameters and 7 prognostic and GCSE episode related factors were defined. Multiple statistical analyses were performed on their relation to cessation markers. *Results*. Onset-to-second-stage-medication (*p* = 0.027), onset-to-burst-suppression (*p* = 0.005), and onset-to-clinical-seizure-freedom (*p* = 0.035) delays correlated with the onset-to-consciousness delay. We detected no correlation between age, epilepsy, STESS, prestatus period, type of SE onset, effect of the first medication, and cessation of SE. *Conclusion*. Our study demonstrates that rapid administration of second-stage medication and early obtainment of clinical seizure freedom and burst-suppression predict early return of consciousness, an unambiguous marker for the end of SE. We propose that delays in treatment chain may be more significant determinants of SE cessation than the previously established outcome predictors. Thus, streamlining the treatment chain is advocated.

## 1. Introduction

Status epilepticus (SE) is a common and life-threatening condition, which requires urgent medical attention. The incidence of SE ranges from 10 to 20 per 100 000 [1] and generalized convulsive SE (GCSE) is by far the most common subtype. SE causes permanent brain damage especially in the hippocampal area [2] and permanently lowers seizure threshold predisposing to epilepsy [3]. Prolonged seizures respond poorly to treatment due to GABA-receptor trafficking occurring along the progressive SE episode [4].

Mortality of SE varies greatly (1.9%–40.0%) in published series [1]. Predictors of a poor outcome include age, structural brain lesion, prolonged seizure, acute symptomatic convulsions, and certain EEG findings during and after SE [5–7]. SE may require treatment in intensive care unit (ICU), which also increases mortality through treatment complications [5, 8]. Both brain damage and mortality are affected by longevity of the seizure and mortality increases greatly after 30 minutes of seizing [9]. In a pediatric study, every minute of ongoing seizure elevated the risk for a seizure prolongation over 60 minutes by five percent [10]. Permanent damage in SE is time-dependent, and seizure duration is the only prognostic factor that can be affected by rapid treatment [11].

It has been recently shown that delays in the treatment of SE are unacceptably long [12]. The effect of treatment delay on prognosis of SE is controversial and subject to debate in the literature. Most studies have focussed on the relation between first-stage medication and outcome, indicated by mortality, patient's condition at discharge and its return to baseline, or by clinical scales. Some studies found that a treatment delay has a clear impact on prognosis; the longer the delay, the worse the outcome [6, 13–15]. A few studies suggested that the treatment delay per se plays an important role on the prognosis besides the etiology [16–18]. Still there are opposite results suggesting that a long treatment delay does not correlate with higher mortality [19–22], and consequently the prognosis of SE is mainly determined by its biological background [23] and affected by its refractoriness [22]. This controversy may partly be explained by the recent observation that, regardless of the adequately started pre-hospital initial treatment, the delays in consecutive parts of the treatment chain were far from optimal [24].

Treatment of SE is guided by two international guidelines [25, 26]. Adherence to treatment protocol, quality of treatment, and management within the recommended time frames seem to have a significant impact on the prognosis of SE [27–29]. Still a recently published study suggested that treatment latency and adherence to protocol were not related to outcome of SE [30].

SE is a very dynamic process with diagnostic challenges, several treatment stages, and potential misinterpretations over the whole management process. Systematic analysis of the factors related to different parts of the treatment chain is needed to draw definite conclusions on the impact of the delays on prognosis. Our newly published study focussed on factors related to delays in the pre-hospital management of SE [24]. To our knowledge, there are no published studies investigating systematically the factors relating to the cessation of SE.

This study was designed to identify the delays and factors related to markers for cessation of GCSE and particularly to identify factors predicting the return of consciousness after GCSE. We also aim at validation of the stepwise definition for the cessation of SE, published earlier [12].

## 2. Methods

### 2.1. Study Design and Setting

This is a retrospective cohort study performed in Helsinki University Central Hospital (HUCH), a tertiary hospital serving a population of 1.4 million. HUCH provides neurological emergency service 24 h a day for the hospital district. The local Emergency Medical Service (EMS) has been instructed to transport patients with GCSE primarily to HUCH Emergency Department (ED). This study conforms to the Finnish legislation concerning medical research and the permission was granted by the HUCH Department of Neurology.

### 2.2. Selection of Participants

This study material includes consecutive adult patients (over 16 years of age) diagnosed with generalized convulsive status epilepticus (GCSE) and treated in the HUCH ED over a two-year period from January 2002 till December 2003.

The patients were identified in the HUCH electronic patient database by the ICD-10 code G41 (SE), yielding a total of 87 patients. Established SE was defined as continuous seizures lasting over 30 minutes, several recurrent seizures without returning consciousness, or occurrence of more than four seizures within any one hour irrespective of return of consciousness in between. Patients not meeting these criteria were excluded, despite having the SE diagnosis in their records, resulting in a total of 82 patients. The seizure description was collected from original medical records for all these patients. Patients having a convulsive seizure at any point of the SE period were considered as having convulsive SE (CSE). Patients with impaired consciousness, either primarily or secondarily, were considered as having generalized SE (GSE). Altogether 70 patients met the criteria for GCSE and were included in this study.

### 2.3. Data Collection

A trained medical doctor collected the data from the original medical records on a standard form designed for this study. The records consisted of notes made by nurses and doctors of EMS, health care centers, regional hospitals, and HUCH ED, ICU, or neurological ward. Ambiguous data were evaluated in collaboration with the research team and if the consensus concerning the original coding rules changed, the data in question were recollected. The electronic database was created using MS Access for data recording. The information of patient identification was removed before further analyses. The Weighted Accuracy Score *L*_WAS_ and the Data Availability (DA) were calculated for all time parameters, using the method developed for evaluation of retrospective delay materials [12]. *L*_WAS_ refers to the deviation of the time parameters from the absolute accuracy in the medical records. The data on delays were based on events with exact time points documented in the medical records, whenever possible. For events not accurately documented, clinically grounded estimation of the event time was based on time frames with exact documented time points at each end.

### 2.4. Measures

Demographic data, medical history of the cases, etiologic and predisposing factors of GCSE and patients' condition at HUCH discharge are presented in Online Table  1 in Supplementary Material available online at http://dx.doi.org/10.1155/2015/591279. Mortality was calculated over HUCH admission period. No postdischarge follow-up was performed in this study.

We defined and calculated 13 parameters for delay in the management of GCSE. All delays were counted from the onset of GCSE. The time parameters and the median delays are presented in Table 1.

We defined 7 grouping variables (prognostic factors and GCSE episode parameters) for subgroup analysis. The variables are presented in Table 2.

Cases with events missing, for example, no burst-suppression (BS) and events happening during prestatus period, or with unknown data were excluded from the final analysis. The missing data information is presented in Online Table  2.

The onset of GCSE was defined as the beginning of the first seizure, fulfilling the criteria for established GCSE. Initial treatment was defined as the first given antiepileptic drug (AED), which was not necessarily the first-stage medication. Alarm delay refers to the primary alarm, in this case the delay in calling the ambulance.

Onset-to-first-convulsion-end refers to the time between the onset of GCSE and the end of the first clinical convulsion. The second-stage medication included i.v. fosphenytoin or valproate, and the third-stage medication included anesthesia with intravenous (i.v.) propofol, thiopental, or midazolam. Induction was considered as the exact time point of anesthesia. First ED was defined as the first emergency department the patient was transported to. Tertiary hospital always refers to HUCH ED.

We defined the markers for cessation of GCSE with three separate parameters for the treatment response [12]: BS, clinical seizure freedom and return of consciousness. BS refers to the beginning of the first BS sequence during this SE. Clinical seizure freedom refers to the end of the last clinical convulsion and return of consciousness refers to the time point, when the patient no longer presented altered mental status.

Age of 65 years was selected as the classification basis for age as a grouping variable. Only patients with previously diagnosed epilepsy were considered as having epilepsy. Status Epilepticus Severity Score (STESS) [21] was calculated for all patients. Seizures occurring no more than 48 h prior to GCSE onset were referred to as the prestatus period seizures. Seizures lasting clinically at least 30 min were defined as continuous. All other types of seizures were considered as intermittent. The patient was considered to respond to the initial treatment, if the seizure stopped within 10 min after i.v. administration or 20 min after rectal administration of the first medication, with no other simultaneous AEDs. Patients failing to respond to the first or second-stage treatment were considered as having refractory SE (RSE). SE continuing or recurring 24 h or more after the onset of anesthesia was considered as super-refractory SE (SRSE).

### 2.5. Statistics

The results are expressed as mean/median and range/interquartile range (IQR) or as number of patients and percentage. The normality of variables was tested with the Kolmogorov-Smirnov test. For the nonnormal data, the Spearman's correlation coefficient and, for normally distributed data, the Pearson's correlation coefficient were calculated to find correlation between continuous variables. Bootstrap resampling (1000 samples) was used to calculate the bias corrected percentile confidence intervals for correlation coefficients. Statistical significance of the differences in variables between independent samples was tested with the nonparametric Wilcoxon-Mann–Whitney test. Differences in categorical variables were examined using the Fisher's exact test. The Kaplan-Meier analysis with the log-rank test was used to analyze time to event data. Linear regression analysis with bootstrap resampling (5000 samples) was used to model delays in treatment response. Statistical analyses were executed using the SPSS software (version 22.0, SPSS, IBM Corp. USA). Statistical significance was defined as *p* < 0.05 and two tailed tests were used.

## 3. Results

The total-time-correlations, that is, correlations between the onset-to-event and onset-to-treatment-response delays, that is, markers for the cessation of SE, are shown in Table 3(a). Since this method includes cumulative delays in the total time from onset to treatment response and therefore may represent inherent correlations, we also calculated the chronological correlations, that is, correlations between the onset-to-event and event-to-treatment-response delays, shown in Table 3(b). Correlation significances given below are based on chronological correlation.

Regardless of the method of calculation, the delays in giving the second-stage medication (*p* = 0.027), obtaining the BS (*p* = 0.005) and achieving the clinical seizure freedom (*p* = 0.035) correlate significantly with the delay in returning of consciousness (Table 3(b)). 76.7% of the BSs were registered with EEG-monitoring before full scale EEG. Therefore, the statistically significant negative correlation between full scale EEG delay and BS delay is clinically insignificant.

**Figure 1:**
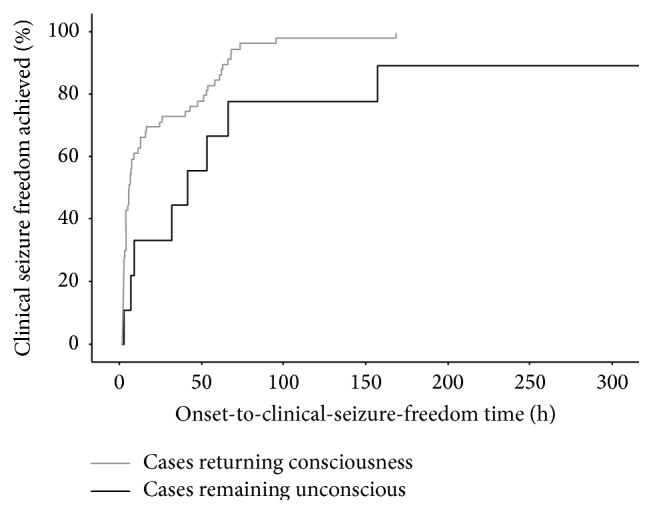
Kaplan-Meier curve showing the difference of the onset-to-clinical-seizure-freedom time between patients returning consciousness and remaining unconscious.

Clinical seizure freedom delay among patients regaining consciousness (*N* = 60, median 3.67 h, 95% CI = 1.64–5.69 h, and DA = 98%, *L*_WAS_ = 1.58) was significantly (*p* = 0.022) shorter than that among patients remaining unconscious (*N* = 9, median 41.17 h, 95% CI 14.87–67.46 h, DA = 100%, and *L*_WAS_ = 1.67) (Figure 1).

The difference in BS delay between patients regaining consciousness (*N* = 22, median 12.0 h, 95% CI 9.32–14.68 h, DA = 95%, and *L*_WAS_ = 1.5) and those remaining unconscious (*N* = 8, median 18.0 h, 95% CI 8.16–27.84 h, DA = 100%, and *L*_WAS_ = 1.5) did not reach statistical significance (*p* = 0.398).

Out of the 70 GCSE cases, 30 cases (42.9%) obtained BS and 40 cases (57.1%) did not. 42 cases (60.0%) of all cases had EEG-monitoring and 30 cases (71.4%) of them obtained BS. In the BS-group eight cases (26.7%) remained unconscious, whereas in the non-BS-group one case (2.5%) remained unconscious, the difference being statistically significant (*p* = 0.004). In the BS-group 23 cases (76.7%) and in the non-BS-group nine cases (22.5%) fulfilled the criteria of SRSE, the difference being statistically significant (*p* < 0.001). The non-BS-group contained all the non-RSE cases of the study material (8/70). Furthermore, in the BS-group all 30 cases were anesthetized with propofol, 20.0% of these cases had multiple anesthetics and 36.7% had several anesthesia periods, the median total anesthesia time being 59 hours 12 minutes (DA = 100%, *L*_WAS_ = 1.48). In the non-BS group all RSE and SRSE cases (32) were anesthetized with only one anesthetic propofol. Four cases (12.5%) had several anesthesia periods, the median total anesthesia time being 20 hours 20 minutes (DA = 97%, *L*_WAS_ = 1.48).

Regression analysis was performed to reveal the correlation of clinical variables with the delays in treatment response. The time parameters having significant effect on the delays of clinical seizure freedom or return of consciousness are shown in Table 4. Regression analysis was not performed on BS delays due to low number of patients (*N* = 30).

Univariate analysis of the factors related to markers for cessation of GCSE is shown in Online Table  3. SRSE cases have significantly longer delays in achieving clinical seizure freedom and returning consciousness than non-SRSE cases (*p* < 0.001).

Univariate analysis of the factors related to return of consciousness is presented in Online Table  4. No significant relations were found, although the non-SRSE cases tended to regain consciousness more likely than the SRSE cases (*p* = 0.070).

In pooled STESS groups 0–2, 42.7% of the cases, and in pooled STESS groups 3–6, 48.6% of the cases presented SRSE. When STESS groups were pooled 0–3 and 4–6, the proportion of cases presenting SRSE was 47.1% and 42.1%, respectively.

## 4. Discussion

This is to our knowledge the first study analysing systematically the delays and factors related to cessation of GCSE. We found that the earlier the clinical seizure freedom is achieved, the earlier and more likely the consciousness returns. Delay of clinical seizure freedom is significantly affected by several delays in the preceding treatment chain. Short delays in giving the second-stage medication and obtaining BS also correlate with early return of consciousness. Surprisingly, several previously reported prognostic factors, such as age, epilepsy, or STESS and the response to initial treatment are related neither to the probability nor the delay of returning consciousness. The present results suggest that the cessation of the GCSE might be more likely related to the delays in the treatment than to the known prognostic factors of SE outcome.

The risk for reporting bias is present in every retrospective study. We controlled the risk by evaluating the adequacy of the data with Data Accuracy (*L*_WAS_) and Data Availability (DA) scores using the method published recently [12]. The scores in this study indicate that the accuracy and coverage of patient data recordings seem to be on an adequate level. We included only patients with GCSE in this study to assure the uniformity of our material.

Although the patient material was collected in 2002-2003 in one tertiary hospital and is relatively small, it is comparable to more recent materials since the treatment recommendations have not markedly changed during the past decade. The increased assortment of intravenously administered second-stage medications in the past years does not affect the interpretation of our results. In this study fosphenytoin was almost exclusively administered as the second-stage medication, providing a relatively homogeneous material. At the time of collection of the material the EEG-monitoring availability was insufficient. Still, the criteria for monitoring and the interpretation of the results have not changed. Direct comparison of the present results to previously published studies should be carried out with caution, since return of consciousness is not widely used in the literature as the marker for cessation of SE. Furthermore, definition of the duration of SE varies considerably among previously published studies. The most commonly used endpoint has been outcome, that is, mortality and/or condition at discharge.

### 4.1. Relation of Delays to Cessation of SE

The exact endpoint of SE is conceptually problematic and varies even in the few previous studies that have clearly defined the endpoint. Cessation of GCSE is defined by Rantsch et al. as the end of the convulsion [22]. Absence of clinical seizure as the only marker seems insufficient since 48% of the seizures continue as electrographic SE [31]. Others have used a combination of last clinical seizure and last continuous electrografic seizure as the criteria without any specific time frames [32, 33]. Mayer et al. used additional time frame criteria, requiring the patient to be seizure free for at least 72 h after the last clinical or electrographic seizure [34]. SE is a dynamic process, and therefore we have suggested a stepwise definition for cessation of SE, including time points of clinical seizure freedom, obtaining BS and return of consciousness [12]. This definition was used in the present study, since return of consciousness is the only reliable clinical marker for the end of GCSE.

Recent evidence indicates that the median delay in giving the second-stage medication and the third-stage medication is nearly the same [12, 24]. The second-stage medication delay resulted mostly from lack of refrigerator in the ambulance required for fosphenytoin storage [12], and in some cases from failure in choosing an adequately specialized hospital for treatment [24]. Two studies have combined fosphenytoin with traditional first-stage treatment given out of hospital [29, 35]. One of them [29] included doctor-led medical emergency teams and the authors found that patients receiving first long-acting AED according to the protocol (fosphenytoin or lorazepam) were 19.9 times more likely to obtain seizure termination (*p* < 0.0001) [29]. In the present study, the delay in giving the second-stage medication showed a significant correlation with the delay in returning consciousness. These results together strongly suggest that the second-stage medication should be available already in EMS units and be administered together with the first-stage medication, provided that adequate physician evaluation of the patient can be obtained to assure the correct diagnosis and the patient safety.

The evidence for the utility of BS as a goal in the treatment of GCSE is scarce and no prospective studies are available. The effect of BS on the prognosis of GCSE patients is controversial [36]. Jaitly et al. found that the presence of BS, regardless of SE etiology or the medication administered, was a sign of a grave prognosis [37]. In another study seizure control without suppression of electric activity to BS or isoelectric level predicted good functional recovery [38]. A few studies have reported that BS had no effect on treatment response or on prognosis [32, 39]. Claassen et al. suggested that BS correlates with favourable treatment response, but still its significance in predicting permanent absence of seizures, mortality, or clinical recovery was questioned [40]. Usefulness of BS as the goal of SE treatment has been advocated based on evidence that the depth of EEG suppression correlates with favourable outcome [41]. Nevertheless, maintenance of BS for at least 24 h is recommended by the European guideline [25], although, as the American guideline states, the EEG endpoint of treatment is controversial, and there are no data indicating as for what duration of treatment is sufficient to obtain permanent seizure termination [26].

In our material the risk of remaining unconscious was significantly higher among patients achieving BS than among patients not treated to BS. The BS-group contained a significantly higher proportion of SRSE cases than the non-BS group. The BS-group also needed more often several anesthetizing agents and repeated anesthesia periods during the total anesthesia time of nearly 40 h longer than that of the non-BS-group. We also showed that the SRSE cases remained unconscious more likely than the non-SRSE cases. We propose that it is not the BS itself that increases the risk of remaining unconscious. Rather, the GCSE of the patients requiring anesthesia to BS seems to be more aggressive than that of the non-BS-group.

No previous studies have focused on the association of BS to the ending of the GCSE or to return of consciousness. We found a significant correlation between early obtainment of BS and early return of consciousness. In our study, the delays in achieving BS did not reach the time frames recommended in the guidelines, reflecting the clinical reality. At least the anesthesia-to-BS delay could be dramatically shortened with an accurate management protocol, as shown in two prospective studies [42, 43]. Since nearly half of our patients seemed to benefit from early BS, the question remains, whether the third-stage treatment given up to BS level in recommended time frames would increase the proportion of patients returning of consciousness also among severe GCSE cases. In parallel, one previous study has speculated that possibly treatment delay is critical for extremely severe SE episodes, although not for all types of SE [20].

We found a significant correlation between early clinical seizure freedom and early return of consciousness. These results are in accordance with the previous literature showing the impact of seizure freedom on prognosis. Claassen et al. found that the delayed seizure control has a negative effect on the efficacy of treatment and that it increases mortality [40]. It has also been stated that the longer the duration of SE the worse the prognosis, particularly after 1-2 h of continuous seizures. It has been claimed that this relation may be lost, if SE lasts over 10 h [36]. However, in our material the mean delay of clinical seizure freedom, although being over 31 h, still correlated with the return of consciousness and thus predicted the cessation of GCSE.

To our surprise onset-to-initial-treatment time, onset-to-diagnosis time, and onset-to-anesthesia time did not correlate to markers for cessation of GCSE. There are no other studies on the effect of diagnosis- and anesthesia-related delays on duration of GCSE in adult patients. There is evidence from a pediatric study suggesting that prehospital diazepam shortens the duration of SE [44]. On the other hand, we have previously shown that short initial treatment delay per se does not lead to a better treatment response delay, unless the whole prehospital treatment chain functions optimally [24].

Regression analysis showed that prolonged time between initial treatment and second-stage treatment predicts a delayed clinical seizure freedom and return of consciousness. Also the time between initial treatment and diagnosis may affect the delay to clinical seizure freedom. Thus, it is feasible that a failure or slow-up in any single delay component may ravage the benefits acquired by optimal action of the earlier phases of the treatment chain.

### 4.2. Relation of Prognostic Factors to Cessation of GCSE

Prognostic factors of SE have been studied in detail, and in most reports the main focus has been on outcome, defined as mortality or clinical status at discharge. To our knowledge, there are no studies on the relation of prognostic factors to cessation of GCSE.

There is a consensus in literature that old age, defined in most studies as the age over 65 years, correlates with worse outcome [21, 36, 45–50]. Significance of age is partly based on pediatric studies, in which age was the major determinant of prognosis, in contrast to adult SE [36]. Prehospital delays in adults do not differ significantly between age groups, when the cutoff point is set at 65 years [24]. In the present study, we did not find any correlation between age and markers for cessation of GCSE. Neither were differences in probability of returning consciousness found between age groups. Our results thus support the previous conclusion that higher mortality of old SE patients may be due to higher frequency of treatment complications [16, 22]. A rational conclusion is that quickly administered second-stage medication should improve the prognosis of GCSE in elderly patients by lessening the need for ICU treatment.

The SE episodes in epilepsy-related cases are commonly thought to be easier to treat, and their outcome is in most studies found to be better than that of patients presenting acute symptomatic seizures [7, 21, 33]. The absence of previous seizures implicates that acute symptomatic seizure as such may be used as a prognostic marker [21]. The delays in the treatment of SE patients with known epilepsy in the prehospital management are shorter than those of the nonepileptic patients. Moreover, epilepsy patients are more likely to be triaged directly to tertiary hospital, diagnosed with SE and anesthetized earlier than nonepileptic patients [24]. Surprisingly, this advantage of shorter delays does not seem to be beneficial to epilepsy patients, suggested by our finding that previously diagnosed epilepsy does not predict faster cessation of GCSE or higher likelihood of returning consciousness.

STESS is an internally and externally validated tool for systematic evaluation of the outcome of SE patients and may be used to recognize the patients who need aggressive treatment [21, 51]. Its scoring criteria include established variables, which all are proven to affect the outcome [21]. However, the cutoff-point for poor outcome is subject to debate [21, 22, 51]. We found no correlation between STESS points and markers for cessation of GCSE with any cutoff. Nor did any STESS point cutoff predict return of consciousness. This is in line with the present observation that two of the STESS variables, age and previous epilepsy diagnosis, lacked correlation with cessation of SE. Taken together, the cessation of GCSE may be more dependent on the management of GCSE than the initial severity of SE.

As can be expected by definition, SRSE cases showed longer delays of clinical seizure freedom and return of consciousness than RSE and non-RSE cases. Interestingly, the delay of obtaining BS did not predict development of SRSE.

### 4.3. Relation of Other Factors to Cessation of GCSE

In our material, the response to first line treatment neither correlated with any of the markers for cessation of GCSE nor predicted return of consciousness. This may be due to the fact that our material included relatively large number of RSE cases, possibly because SE cases successfully treated with first-stage medication were not referred to the tertiary hospital. A recent study reported that the efficacy of the first-stage treatment does not affect the duration of SE [52], while another study presented an opposite result [35].

Pre-SE period, that is, occurrence of recurring convulsive seizures preceding the actual SE, is a newly introduced concept [12], the significance of which has not yet been established. In the present study, pre-SE period did not correlate with the delay in cessation of SE. We did not find any significant correlation between the type of SE onset and return of consciousness.

## 5. Conclusions

We conclude that early administration of second-stage medication, early cessation of clinical seizures, and early obtainment of BS predict early return of consciousness, which is an unambiguous marker for the cessation of SE. The present retrospective study suggests that delays in treatment chain may be more significant determinants of SE cessation than the previously established outcome predictors. The correlations presented here serve as validation for the use of stepwise definition of the end of SE and speak for consideration of BS as the target of the third-stage treatment. The delays should also be considered in planning protocols, particularly in matching of patient groups, in prospective SE studies.

## Supplementary Material

Table 1. Description of the material.
Table 2. Detailed information of the missing data of the 70 consecutive GCSE patients.
Table 3.Univariate analysis of the factors related to markers for cessation of GCSE.
Table 4. Univariate analysis of the factors relation to returning of consciousness.

## Figures and Tables

**Table 1:** Delay parameters and the delays in the management of GCSE.

Variable	*N*	%	Time	Time	MIN	MAX	DA	*L*_WAS_
All Cases	70	100	Median	Mean			%	
Delays in the treatment								
Onset-to-initial-treatment	67	95.7	30 min	57 min	0 min	8 h 5 min	97.0	1.8
Onset-to-alarm	60	85.7	36 min	2 h 27 min	0 min	57 h 44 min	93.3	1.5
Onset-to-first-convulsion-end	70	100	51 min	2 h 13 min	1 min	63 h 40 min	97.1	1.8
Onset-to-diagnosis	70	100	1 h 48 min	4 h	6 min	60 h 6 min	97.1	1.5
Onset-to-second-stage-medication	67	95.7	2 h 40 min	4 h 49 min	30 min	61 h 54 min	98.5	1.6
Onset-to-anesthesia	62	88.6	2 h 38 min	5 h 43 min	0 min	66 h 20 min	98.4	1.5
Onset-to-first-ED	61	87.1	2 h 2 min	3 h 31 min	0 min	58 h 29 min	98.4	1.5
Onset-to-tertiary-hospital (HUCH)	70	100	2 h 25 min	1 h 25 min	37 min	277 h 40 min	98.6	1.5
Onset-to-EEG	57	81.4	21 h 52 min	33 h	2 h 30 min	142 h	94.7	1.5
Onset-to-EEG-monitoring	42	60.0	11 h 10 min	15 h 45 min	2 h 30 min	82 h 14 min	97.6	1.5
Delays in the markers for cessation of GCSE								
Onset-to-burst-suppression	30	42.9	14 h 42 min	25 h 20 min	5 h 5 min	137 h 50 min	100.0	1.5
Onset-to-clinical-seizure-freedom	70	100	5 h 15 min	31 h 5 min	26 min	533 h 15 min	98.6	1.6
Onset-to-consciousness	61	87.1	42 h 45 min	66 h 5 min	2 h 40 min	444 h 40 min	96.7	1.4

**Table 2:** Grouping variables (prognostic factors and GCSE episode parameters) for the subgroup analysis.

Variable	*N*	%
All	70	100
Age under 65		
Yes	51	72.9
No	19	27.1
Epilepsy		
Yes	46	65.7
No	23	32.9
Unknown	1	1.4
STESS		
2	35	50.0
3	16	22.9
4	10	14.3
5	9	12.9
Prestatus period		
Yes	14	20.0
No	56	80.0
SE onset		
Continuous	45	64.3
Intermittent	25	35.7
Effect of the first medication		
Yes	17	24.3
No	39	55.7
Spontaneous cessation	11	15.7
Refractoriness		
Non-RSE	8	11.4
RSE	30	42.9
SRSE	32	45.7

**(a):** 

Variable	Onset-to-burst-suppression					Onset-to-clinical-seizure-freedom					Onset-to-consciousness				
	*N*	Coefficient	95% CI (min)	95% CI (max)	*p* value	*N*	Coefficient	95% CI (min)	95% CI (max)	*p* value	*N*	Coefficient	95% CI (min)	95% CI (max)	*p* value
Onset-to-initial-treatment	28	0.127	−0.299	0.519	0.520	65	0.054	−0.181	0.289	0.668	56	0.017	−0.213	0.254	0.902
Onset-to-first-convulsion-end	29	0.303	−0.093	0.640	0.110	68	0.122	−0.122	0.363	0.321	58	0.107	−0.166	0.367	0.425
Onset-to-alarm	22	0.467	0.036	0.791	**0.029**	55	0.300	−0.004	0.558	**0.026**	47	0.010	−0.256	0.288	0.946
Onset-to-diagnosis	29	0.415	0.040	0.691	**0.025**	68	0.371	0.120	0.587	**0.002**	59	0.136	−0.111	0.390	0.304
Onset-to-second-stage-medication	30	0.337	−0.080	0.660	0.069	66	0.334	0.087	0.535	**0.006**	56	0.402	0.165	0.610	**0.002**
Onset-to-anesthesia	30	0.510	0.132	0.765	**0.004**	61	0.382	0.098	0.619	**0.002**	51	0.088	−0.175	0.353	0.540
Onset-to-first-ED	23	0.500	0.091	0.781	**0.015**	60	0.260	0.019	0.503	**0.045**	52	0.212	−0.740	0.476	0.131
Onset-to-tertiary-hospital (HUCH)	30	0.538	0.177	0.780	**0.002**	69	0.334	0.081	0.559	**0.005**	59	0.207	−0.550	0.448	0.116
Onset-to-EEG	26	0.099	−0.324	0.496	0.632	54	0.470	0.197	0.688	**<0.001**	46	0.327	0.037	0.568	**0.027**
Onset-to-EEG-monitoring	30	0.775	0.503	0.929	**<0.001**	41	0.413	0.096	0.677	**0.007**	31	0.239	−0.135	0.542	0.194
Onset-to-burst-suppression						30	0.558	0.165	0.857	**0.001**	21	0.527	0.071	0.815	**0.014**
Onset-to-clinical-seizure-freedom											59	0.739	0.576	0.850	**<0.001**

**(b):** 

Variable	Event-to-burst-suppression					Event-to-clinical-seizure freedom					Event-to-consciousness				
Onset-to-event	*N*	Coefficient	95% CI (min)	95% CI (max)	*p* value	*N*	Coefficient	95% CI (min)	95% CI (max)	*p* value	*N*	Coefficient	95% CI (min)	95% CI (max)	*p* value
Onset-to-initial-treatment	28	0.005	−0.421	0.412	0.981	65	−0.095	−0.344	0.156	0.453	56	−0.012	−0.237	0.205	0.928
Onset-to-first-convulsion-end	29	0.109	−0.282	0.474	0.573	68	−0.112	−0.360	0.136	0.362	58	0.085	−0.173	0.322	0.528
Onset-to-alarm	22	0.303	−0.176	0.666	0.171	55	0.020	−0.253	0.295	0.883	47	−0.087	−0.364	0.223	0.563
Onset-to-diagnosis	29	0.169	−0.198	0.497	0.382	68	−0.069	−0.321	0.226	0.574	59	0.037	−0.267	0.322	0.783
Onset-to-second-stage-medication	30	0.057	−0.345	0.421	0.765	66	−0.046	−0.323	0.265	0.713	56	0.295	0.039	0.534	**0.027**
Onset-to-anesthesia	30	−0.152	−0.488	0.175	0.424	61	−0.057	−0.333	0.211	0.662	51	0.025	−0.251	0.330	0.859
Onset-to-first-ED	23	0.343	−0.062	0.732	0.109	60	−0.022	−0.296	0.271	0.870	52	0.101	−0.195	0.385	0.477
Onset-to-tertiary-hospital (HUCH)	30	0.113	−0.247	0.498	0.552	69	−0.037	−0.285	0.195	0.761	59	0.068	−0.220	0.338	0.610
Onset-to-EEG	26	−0.753	−0.914	−0.473	**<0.001**	54	−0.198	−0.475	0.081	0.152	46	−0.162	−0.420	0.116	0.283
Onset-to-EEG-monitoring	30	−0.183	−0.579	0.278	0.332	41	−0.051	−0.386	0.279	0.752	31	0.101	−0.311	0.459	0.588
Onset-to-burst-suppression						30	0.031	−0.359	0.443	0.872	21	0.584	0.058	0.863	0.005^*∗*^
Onset-to-clinical-seizure-freedom											59	0.275	−0.036	0.563	**0.035**

Spearman's rho.

^*∗*^Pearson's rho.

**Table 4:** The regression analysis of the effect of the chronological delay components on markers for cessation on GCSE.

Variable	TIME	95% CI	95% CI	*p*
	(h)	min	max	
Onset-to-clinical-seizure-freedom				

Intercept	9.0	−1.4	23.6	0.082
Onset-to-initial-treatment	7.8	−1.6	13.2	0.008
Initial-treatment-to-diagnosis	2.3	0.2	4.2	**0.016**

Intercept	8.2	−5.6	31.3	0.273
Onset-to-initial-treatment	6.6	−2.8	11.6	0.035
Initial-treatment-to-second-stage-medication	3.0	0.4	4.8	**0.021**

Onset-to-consciousness				

Intercept	38.1	14.8	73.4	0.008
Onset-to-initial-treatment	0.4	−15.2	8.1	0.935
Initial-treatment-to-second-stage-medication	9.7	3.9	15.8	**0.002**
