# Supplementary material for: Delays and Factors Related to Cessation of Generalized Convulsive Status Epilepticus
Source: Epilepsy Res Treat. 2015 Aug 10;2015:591279. doi: 10.1155/2015/591279 (PMC4546976; doi:10.1155/2015/591279)
Supplement: Supplementary file 1 — Table 1. Description of the material. Table 2. Detailed information of the missing data of the 70 consecutive GCSE patients. Table 3.Univariate analysis of the factors related to markers for cessation of GCSE. Table 4. Univariate analysis of the factors relation to returning of consciousness. [file 591279.f1.pdf]

ONLINE TABLE 1.

Description of the material.

ONLINE TABLE 2.

Detailed information of the missing data of the 70 consecutive GCSE patients.

ONLINE TABLE 3.

Univariate analysis of the factors related to markers for cessation of GCSE.

ONLINE TABLE 4.

Univariate analysis of the factors relation to returning of consciousness.

ONLINE TABLE 1.

VARIABLE	N	%
**Cases**	70	100
**Age**		
Mean	54.3	
Range	16-85	
**Gender**	70	100.0
Male	35	50.0
Female	35	50.0
**Medical history**		
Previous recorded illnesses	70	100.0
Epilepsy	46	65.7
**Etiologies**		
Epilepsy	46	65.7
Acute brain disorder	7	10.0
Prior brain disorder	7	10.0
Unknown	10	14.3
**Predisposing factors**	42	60.0
Alcohol	11	15.7
Inappropriate epilepsy medication	16	22.9
Physical or emotional stress	7	10.0
Electrolyte disorder	6	8.6
Infection	5	7.1
Brain oedema	2	2.9
Sleep deprivation	2	2.9
Other	8	11.4
**Condition at HUCH discharge**		
Conscious	60	85.7
Unconscious	5	7.1
Dead	5	7.1

ONLINE TABLE 2

VARIABLE	EVENT DURING PRE-STATUS PERIOD	EVENT MISSING	DATA UNKNOWN	DATA INACCURATE
**DELAYS IN THE TREATMENT**				
Onset-to-initial-treatment	3	-	-	2
Onset-to-alarm	6	6	-	3
Onset-to-first-convulsion-end	-	-	-	2
Onset-to-diagnosis	-	-	-	2
Onset-to-second-stage-medication	-	3	-	1
Onset-to-anesthesia	-	8	-	1
Onset-to-first-ED	2	7	-	1
Onset-to-tertiary-hospital (HUCH)	-	-	-	1
Onset-to-EEG	-	13	-	3
Onset-to-EEG-monitoring	-	28	-	1
**MARKERS FOR CESSATION OF GCSE**				
Onset-to-Burst-suppression	-	40	-	0
Onset-to-clinical-seizure-freedom	-	-	-	1
Onset-to-consciousness	-	9	-	2
**SUBGROUP VARIABLES**				
Age under 65	-	-	-	-
Epilepsy	-	-	1	-
STESS	-	-	-	-
Pre-status period	-	-	-	-
SE onset	-	-	-	-
Effect of the first medication	3	-	-	-
Refractoriness	-	-	-	-

ONLINE TABLE 3.

VARIABLE		N	%	Time	Min	Max	DA	L_{WAS}	P-value
ALL		70	100	Median			%		
Age under 65 (Yes/No)									
Onset-to-clinical-seizure-freedom									
	Yes	51	72.9	6h	32min	533h 15min	100.0	1.56	0.12
	No	19	27.1	2h 58min	26min	66h 1min	94.7	1.67	
Onset-to-burst-suppression									
	Yes	22	31.4	14h 43min	5h 30min	51h 30min	100.0	1.45	0.81
	No	8	11.4	15h	5h 5min	137h 50min	100.0	1.63	
Onset-to-consciousness									
	Yes	45	64.3	42h 45min	2h 40min	444h 40min	100.0	1.42	0.84
	No	16	22.9	35h 38min	3h 5min	182h 30min	87.5	1.43	
Epilepsy (Yes/No/Unknown)									
Onset-to-clinical-seizure-freedom									
	Yes	46	65.7	4h 48min	32min	533h 15min	97.8	1.61	0.50
	No	23	32.9	6h 30min	26min	72h 20min	100.0	1.54	
Onset-to-burst-suppression									
	Yes	19	27.1	14h 55min	5h 5min	137h 50min	100.0	1.55	0.99
	No	10	14.3	16h 15min	9h 50min	75h	100.0	1.41	
Onset-to-consciousness									
	Yes	42	60.0	30h 30min	2h 40min	444 40min	95.2	1.50	0.61
	No	18	25.7	50h 53min	3h 5min	122h 30min	100.0	1.26	
STESS (0-2/3-6)									
Onset-to-clinical-seizure-freedom									
	0-2	35	50.0	6h	32min	533h 15min	100.0	1.57	0.43
	3-6	35	50.0	3h 40min	26min	72h 20min	97.1	1.60	
Onset-to-burst-suppression									
	0-2	14	20.0	15h 58min	5h 30min	51h 30min	100.0	1.46	0.50
	3-6	16	22.9	13h 30min	5h 5min	137h 50min	100.0	1.53	
Onset-to-consciousness									
	0-2	31	44.3	32h 30min	2h 40min	444h 40min	100.0	1.48	0.62
	3-6	30	42.9	44h 40min	3h 5min	182h 30min	93.3	1.36	
STESS (2/3/4/5)									
Onset-to-clinical-seizure-freedom									
	2	35	50.0	6h	32min	533h 15min	100.0	1.57	0.18
	3	16	22.9	5h 38min	1h 5min	72h 20min	100.0	1.53	
	4	10	14.3	1h 30min	32min	50h 45min	90.0	1.83	
	5	9	12.9	6h 30min	26min	66h 1min	100.0	1.50	
Onset-to-burst-suppression									
	2	14	20.0	15h 58min	5h 30min	51h 30min	100.0	1.46	0.75
	3	8	22.4	13h 30min	9h 50min	28h 47min	100.0	1.44	
	4	5	7.1	12h	5h 5min	137h 50min	100.0	1.80	
	5	3	4.3	18h	11h	75h	100.0	1.33	

Onset-to-consciousness								
2	31	44.3	32h 30min	2h 40min	444h 40min	100.0	1.48	0.89
3	14	20.0	50h 2min	14h 30min	146h 30min	100.0	1.29	
4	10	14.3	25h 23min	10h	182h 30min	80.0	1.63	
5	6	8.6	53h 5min	3h 5min	122h 30min	100.0	1.17	
Pre-status period (Yes/No)								
Onset-to-clinical-seizure-freedom								
Yes	14	20.0	4h 28min	45min	533h 15min	100.0	1.54	0.98
No	56	80.0	5h 45min	26min	94h 15min	98.2	1.60	
Onset-to-burst-suppression								
Yes	8	11.4	12h 15min	9h 50min	50h 50min	100.0	1.44	0.67
No	22	31.4	17h 30min	5h 5min	137h 50min	100.0	1.52	
Onset-to-consciousness								
Yes	12	17.1	40h 23min	9h 45min	294h	100.0	1.33	0.87
No	49	70.0	45h 15min	2h 40 min	444h 40min	95.9	1.45	
SE onset (Continuous/Intermittent)								
Onset-to-clinical-seizure-freedom								
Continuous	45	64.3	5h 53min	32min	156h 42min	97.8	1.56	0.58
Intermittent	25	35.7	4h 15min	26min	533h 15min	100.0	1.64	
Onset-to-burst-suppression								
Continuous	19	27.1	18h	5h 5min	137h 50min	100.0	1.44	0.38
Intermittent	11	15.7	14h	6h 10min	41h	100.0	1.64	
Onset-to-consciousness								
Continuous	40	57.1	47h 40min	2h 40min	444h 40min	95.0	1.39	0.15
Intermittent	21	30.0	25h 30min	3h 5min	181h 45min	100.0	1.48	
Effect of the 1. medication (Yes/No/Spont.cessation/1.med.prior SE)								
Onset-to-clinical-seizure-freedom								
Yes	17	24.3	3h 40min	50min	66h 30min	100.0	1.59	0.62
No	39	55.7	11h 10min	32min	156h 42min	100.0	1.54	
Spont. cessation	11	15.7	3h 44min	26min	166h 45min	90.9	1.70	
1. med.prior SE	3	4.3	6h	4h 40min	533h 15min	100.0	1.83	
Onset-to-burst-suppression								
Yes	6	8.6	11h 15min	9h	22h 1min	100.0	1.58	0.40
No	17	24.3	21h 10min	5h 5min	137h 50min	100.0	1.38	
Spont. cessation	5	7.1	17h	11h	41h	100.0	1.90	
1. med.prior SE	2	2.9	13h 28min	12h	14h 55min	100.0	1.25	
Onset-to-consciousness								
Yes	14	20.0	21h 17min	2h 40 min	71h	100.0	1.39	0.87
No	35	50.0	55h 30min	3h 50min	444h 40min	97.1	1.43	
Spont. cessation	10	14.3	28h 30min	3h 5min	172h	90.0	1.44	
1. med.prior SE	2	2.9	50h 23min	42h 45min	58h	100.0	1.50	
Refractoriness (Non-RSE/RSE/SRSE)								
Onset-to-clinical-seizure-freedom								
Non-RSE	8	11.4	1h 53min	32min	59h 19min	100.0	1.63	**<0.001**
RSE	30	42.9	2h 35min	26min	94h 15min	96.7	1.53	

	SRSE	32	45.7	43h 55min	50min	533h 15min	100.0	1.63	
Onset-to-burst-suppression									
	Non-RSE	0	0					0.00	0.33
	RSE	7	10.0	11h	5h 5min	28h 30min	100.0	1.50	
	SRSE	23	32.9	18h	6h 10min	137h 50min	100.0	1.50	
Onset-to-consciousness									
	Non-RSE	8	11.4	9h 53min	2h 40min	89h 20min	100.0	1.56	**<0.001**
	RSE	29	41.4	22h 20min	3h 5min	94h 30min	93.1	1.39	
	SRSE	24	34.3	84h 8min	42h 45min	444h 40min	100.0	1.42	
Refractoriness (Non-SRSE/ SRSE)									
Onset-to-clinical-seizure-freedom									
	Non-SRSE	38	54.3	2h 30min	26min	94h 15min	97.4	1.55	**<0.001**
	SRSE	32	45.7	43h 55min	50min	533h 15min	100.0	1.63	
Onset-to-burst-suppression									
	Non-SRSE	7	10.0	11h	5h 5min	28h 30min	100.0	1.50	0.31
	SRSE	23	32.9	18h	6h 10min	137h 50min	100.0	1.50	
Onset-to-consciousness									
	Non-SRSE	37	52.9	20h 40min	2h 40min	94h 30min	94.6	1.56	**<0.001**
	SRSE	24	34.3	84h 8min	42h 45min	444h 40min	100.0	1.43	

ONLINE TABLE 4

VARIABLE		ALL	Conscious		Unconscious		P-values
		70	N	%	N	%	Fisher p
Age under 65							
	Yes	51	45	88.2	6	11.8	0.696
	No	19	16	84.2	3	15.8	
Epilepsy							
	Yes	46	42	91.3	4	8.7	0.148
	No	23	18	78.3	5	21.7	
	Unknown	1					
STESS							
	2	35	31	88.6	4	11.4	0.198
	3	16	14	87.5	2	12.5	
	4	10	10	100.0	0	0	
	5	9	6	66.7	3	33.3	
STESS							
	0-2	35	31	88.6	4	11.4	1.000
	3-6	35	30	85.7	5	14.3	
STESS							
	0-3	51	45	88.2	6	11.8	0.696
	4-6	19	16	84.2	3	15.8	
Pre-status period							
	Yes	14	12	85.7	2	14.3	1.000
	No	56	49	87.5	7	12.5	
SE onset							
	Continuous	45	40	88.9	5	11.1	0.712
	Intermittent	25	21	84.0	4	16.0	
Effect of the first medication							
	Yes	17	14	82.4	3	17.6	0.515
	No	39	35	89.7	4	10.3	
	Spontaneous cessation	11	10	90.9	1	9.1	
	1.med before SE	3	2	66.7	1	33.3	
Refractoriness							
	Non-RSE	8	8	100.0	0	0	0.151
	RSE	30	28	93.3	2	6.7	
	SRSE	32	25	78.1	7	21.9	
Refractoriness							
	Non-RSE	8	8	100.0	0	0	0.584
	RSE/SRSE	62	53	85.5	9	14.5	
Refractoriness							
	Non-SRSE	38	36	94.7	2	5.3	0.070
	SRSE	32	25	78.1	7	21.9	
